# Motivational profiles, accelerometer-derived physical activity, and acute diabetes-related symptoms in adults with type 2 diabetes

**DOI:** 10.1186/s12889-018-5376-y

**Published:** 2018-04-10

**Authors:** Alexandre Castonguay, Paule Miquelon

**Affiliations:** Department of Psychology, Université du Québec à Trois-Rivières, 3351, boul. des Forges, C.P. 500, Trois-Rivières, QC G9A 5H7 Canada

**Keywords:** Type 2 diabetes, Physical activity behavior, Accelerometer, Self-determination theory, Motivational profile, Diabetes-related symptoms

## Abstract

**Background:**

Using self-determination theory, the objective of this study was to examine, over a one-month period, how physical activity (PA) motivation would influence accelerometer-derived PA behavior, and ultimately, acute diabetes-related symptoms burden among adults with type 2 diabetes (T2D adults). Using both a person and variable-centered approach, this objective was attained by means of: 1) investigating the indirect effect of PA participation on the relationship between PA motivation and acute diabetes-related symptom burden and 2) examining whether participants who met PA recommendations (i.e., 150 min of moderate-to-vigorous PA per week) would experience less acute diabetes-related symptom burden over a one-month period.

**Methods:**

A two-wave prospective longitudinal design was used. At time 1, participants completed a questionnaire assessing their PA motivation and were asked to wear an ActiGraph GT3x accelerometer for four consecutive weeks. At time 2, they completed a short questionnaire assessing their acute diabetes-related symptoms (i.e., symptoms related to fatigue, cognitive distress, hyperglycemia, and hypoglycemia). The final sample includes 165 adults (89 or 53.61% women) aged from 26 to 75 years (M = 62.05, SD = 8.75) with T2D, which provided at least 21 valid days of accelerometer-derived data.

**Results:**

First, results of a path analysis demonstrated that over a one-month period, the average number of minutes spent practicing moderate to vigorous PA per week mediated the relationship between intrinsic and external PA motivation and the level of burden associated with the following diabetes-related symptoms: fatigue, cognitive distress, and hyperglycemia. In addition, results of covariance analyses showed that participants meeting PA recommendations also reported significantly less burden associated with these three symptoms over a month period. Then, the existence of four motivational profiles (Self-Determined, High Introjected, Low Motivation, and Non-Self-Determined) was confirmed using a k-means analysis. Results of covariance and chi-square analyses further showed, respectively, that compared to other motivational profiles, the Self-Determined profile was associated with a higher score on weekly PA participation and meeting PA recommendations.

**Conclusions:**

The results highlight the importance of promoting autonomous motives for PA participation among T2D adults. They also suggest that T2D adults meeting PA recommendations experience less acute diabetes-related symptoms burden, which further support the importance of their PA motivation.

## Background

Regular physical activity (PA) is crucial to managing type 2 diabetes (T2D) as it improves glycemic control, decreases insulin resistance, improves the lipid profile, reduces blood pressure, and facilitates weight loss [1–4]. However, many individuals with diabetes are insufficiently active and do not meet the recommendation of 150 min of moderate-to-vigorous PA (MVPA) per week, as proposed by the Canadian Diabetes and the American Diabetes Associations [4, 5]. For instance, between 61% and 65% of adults with diabetes (among which 90% are estimated to have T2D) reported being insufficiently active in the United States and Canada [6–8].

These participation trends show it has become essential to understand why so few adults with T2D (hereafter T2D adults) regularly practice PA, despite its beneficial effects on their health. Therefore, studying the quality of motivations associated with PA seems highly relevant, as among the PA barriers identified by individuals with T2D, a lack of motivation and enjoyment are determining factors [9]. A highly promising theoretical approach to understanding how motivation influences the adoption and maintenance of PA is self-determination theory (SDT) [10]. SDT adopts a multi-dimensional approach to explain why some individuals and not others engage in positive and adaptive health behaviors by examining the extent to which a person’s motivation for a particular behavior is autonomous or controlled. Based on the autonomy and personal choice associated with one’s behavior, SDT proposes conceptualizing motivation along a continuum ranging from amotivation to controlled motivation to autonomous motivation. Amotivation refers to a lack of intention to act, and it is characterized by engaging in actions without any motivation. Amotivated individuals might say they avoid PA because they find it uninteresting. Controlled motivation is less autonomous, and it includes external and introjected regulations. External regulation involves acting to satisfy an external demand or receive a reward. For instance, individuals could engage in PA based only on their physician’s recommendation. Introjected regulation involves internalizing a regulation but not accepting it as one’s own, such as acting to avoid guilt or enhance the ego. In this case, individuals could act to avoid negative feelings (e.g., guilt) or because they want to prove themselves.

Autonomous motivation comprises identified and intrinsic regulations. Identified regulation occurs when individuals engage in an activity they consider personally valuable and important to obtaining a desired outcome. In this case, the person endorses the behavior and performs it with a high degree of perceived autonomy. Hence, identified regulation is considered an autonomous form of motivation because it reflects a sense of personal volition. For example, one may engage in PA because he/she considers it a good way to maintain his/her health, which represents valuable outcomes for him/her. The person exercises for the usefulness or importance of exercise (e.g., to be healthy). Finally, the most autonomous motivation type is intrinsic regulation, where people are intrinsically motivated to practice PA because it is inherently enjoyable.

### Research using SDT to examine PA motivation among T2D adults

To date, many studies have examined the relationship between motivation and PA in the general population, showing consistent support for a positive relationship between autonomous motives and several PA outcomes, including PA frequency and duration [11]. However, fewer SDT-based studies have looked at the association between motivation and PA in the adult T2D population [12–19]. Although these studies have shown that autonomous motivation (assessed using a global score) is positively associated with various positive PA outcomes, only two studies have thus far investigated the respective impact of each motivation type (including controlled types) on PA behavior, including observing PA recommendations for T2D (i.e., accumulating at least 150 min of MVPA/week) [17, 18]. More precisely, Miquelon and Castonguay [17, 18] reported the relationship between each motivation type and PA behavior, as measured subjectively. As a whole, their results support SDT assumptions. In the study published by Miquelon and Castonguay [17], PA participation was associated positively with intrinsic and identified regulations, while it was associated negatively with external regulation and amotivation. In addition, Castonguay and Miquelon [18] reported similar and significant associations between each motivation type and subjective PA participation.

Of note is that among the few SDT-based studies that surveyed the association between motivation and PA in a T2D adult population, only two have analyzed motivational profiles and their relationship with PA participation among T2D adults [18, 19]. Compared to a variable-centered approach (i.e., examining the effects of each motivation type on a specific outcome), assessing motivational profiles increases the understanding of how different motivational regulations coexist in individuals [20–22]. Considering motivational profiles is important because although individuals might, for instance, engage in PA because of external pressures, they might also enjoy the benefits of the activity, performing it for both reasons. Using a person-centered approach by assessing how different motivation types combine to form motivational profiles is hence a promising way to account for individual motivational configurations [23, 24]. Overall, the results obtained by these two studies revealed that motivational profiles benefit from higher autonomous motivation levels and lower controlled motivation levels regarding PA participation. Specifically, Gourlan et al. [19] found three motivational profiles: Self-determined Determined (high intrinsic, integrated, and identified regulation scores; low scores for other regulations), High Combined (high motivation scores for intrinsic to external regulations, moderate amotivation scores), and Moderate (moderate scores for all regulations). They demonstrate that compared to moderate participants, high combined and self-determined participants reported practicing PA for more hours/week over the past 12 months. For their part, Castonguay and Miquelon [18] found four motivational profiles: Self-Determined (higher intrinsic and identified regulation scores, lower external regulation and amotivation scores), Moderate (moderate scores for all motivations), Controlled (lower intrinsic, identified, and amotivation scores; higher introjected and external regulation scores), and Non-Self-Determined (higher amotivation scores, moderate introjected and external regulation scores, lower intrinsic and identified regulation scores). Their results showed that participants from the Self-Determined profile who reported engaging in PA for more hours/week over the past three months spent more time practicing MVPA per week than participants from other profiles. Although both studies offer valuable knowledge regarding PA motivational profiles among T2D adults, additional research is required to investigate this issue further, as both studies relied on a cross-sectional design and PA self-report measure to verify the relationship between PA motivational profiles and PA behavior.

### PA behavior and diabetes-related symptoms

The impact of PA on glycemic control, assessed by means of HbA1c, is well documented among adults with T2D [25–27]. However, HbA1c reflects glycemic control over a three-months period and, in the context of shorter-duration research, only acute symptoms (i.e., fatigue, cognitive distress, hypoglycemia, and hyperglycemia) resulting from fluctuating blood sugars are expected to show immediate change [28]. Therefore, these acute diabetes-related symptoms can be used to detect changes resulting from fluctuating blood sugars over a short period. Though prior research has surveyed the influence of PA on glycemic control using the HbA1c indicator, the influence of PA participation on the experience of diabetes-related symptoms, including acute ones, has been investigated much less, if at all. Thus, the relationship between PA participation, along with meeting PA recommendations, and acute diabetes-related symptoms still has to be investigated.

### Objectives of the present study

This study addresses an important limitation of prior SDT-based studies, including interventional ones, examining the relationship between motivation and PA behavior among adults with T2D, that is, the exclusive reliance on self-reported measures to asses PA behavior. In fact, thus far, the relationship between motivation, as conceptualized by SDT, and accelerometer-derived PA has been evaluated in different populations (e.g., adolescents, the elderly, and women) [29–38], but not in adults with T2D. Worth mentioning is that, although the results of these aforementioned studies support a positive relationship between autonomous motivation and accelerometer-derived PA behavior, most have not considered the respective impact of each motivation on accelerometer-derived PA behavior; rather, they used an autonomous motivation variable or the relative autonomy index [34–38]. Moreover, of these studies, few have reported more than one week of objectively measured PA participation [32, 37], with only one reporting at least three weeks (23 days) [37]. Most of these studies required participants to wear the accelerometer for seven consecutive days, and they reported objective PA measures for a period of four to seven days. Finally, only two of these studies examined the relationship between meeting PA recommendations (i.e., cumulating at least 150 min of MVPA/week) and autonomous motivation [31, 36], which was positive.

In regards of the limitations outlined above, it becomes relevant to explore the respective impact of each motivation type, and motivational profiles, on accelerometer-derived PA behavior among T2D adults. The findings of such exploratory research could provide an empirical basis for future interventional studies looking for ways to promote PA participation through PA motivation among T2D adults. Therefore, the main objective of the herein study, which uses a prospective longitudinal design, is to evaluate, over a one month-period, the impact of PA motivation (using both a person- and variable-centered approach) on accelerometer-derived PA behavior, assessed by the average number of minutes spent practicing MVPA per week, in a sample of adults with T2D. The average number of minutes spent practicing MVPA per week was chosen to assess PA behavior given that most prior SDT-based studies surveying accelerometer-derived PA behavior have used it as their main PA indicator [29, 31–36, 38]. Moreover, as the core reason underlying PA promotion among T2D adults is achieving better glycemic control and, hence, experiencing less diabetes-related symptoms (e.g., fatigue and hypoglycemia), an additional aim is to examine the indirect effect of PA motivation on the experience of diabetes-related symptoms burden through accelerometer-derived PA behavior. As aforementioned, in the context of shorter-duration research, such as the herein one, covering a one month-period, merely acute diabetes-related symptoms resulting from fluctuating blood sugars are expected to show rapid change. For that reason, only acute diabetes-related symptoms are measured in the present study. Finally, a third aim is to verify whether participants who meet PA recommendations (i.e., engaging in at least 150 min of moderate-to-vigorous PA per week) experience less acute diabetes-related symptom burden over a one-month period.

## Methods

### Participants and procedure

A two-wave prospective longitudinal design was used, and participants were recruited through Diabetes Quebec, a non-profit association that informs and supports people with diabetes. Potential participants were T2D adults aged between 18 and 75 years who were solicited randomly across Quebec to participate in the study. In total, 201 participants (94 men and 106 women, mean age = 61.85 years, standard deviation [SD] = 8.80) agreed to partake in the study. They were asked to provide written informed consent and complete two web-based surveys. The time 1 (T1) survey assessed participants’ demographic information (i.e., age, gender, diabetes type (1 or 2), number of years since T2D diagnosis, and height and weight to calculate their BMI) and PA motivation, while the time 2 (T2) survey, completed a month later, evaluated their acute diabetes-related symptoms. Participants were also asked to wear an ActiGraph (Pensacola, FL) GT3X accelerometer on an elastic waistband for four consecutive weeks. Although 201 adults initially completed the survey, the analyses are based on 165 participants’ data. The sample size was reduced for numerous reasons: two participants who indicated they had type 1 diabetes were excluded, two were aged over 75 years, 13 reported a health condition that would greatly reduce their PA practice (e.g. fibromyalgia, major injury, paralysis, etc.), and seven participants did not answer all required questions. Moreover, as only the data from participants who wore the ActiGraph for at least 21 days were analyzed, 10 additional participants were excluded. Finally, because a cluster analysis is sensitive to multivariate outliers [39], one case was also excluded from the analyses. The final sample thus comprised 165 participants (89 or 53.61% women) aged from 26 to 75 years (M = 62.05, SD = 8.75)^1^ living with T2D for approximately 11.42 years (SD = 8.19) and who had a mean body mass index (BMI) of 30.74 (SD = 6.47).

### Measures

#### PA motivation

The Behavioral Regulation in Exercise Questionnaire-version 2 (BREQ-2) [40] was used to measure PA motivations. It includes five subscales assessing intrinsic (e.g., “I enjoy my exercise sessions;” *n* = 4), identified (e.g., “It’s important to me to exercise regularly;” *n* = 4), introjected (e.g., “I feel guilty when I do not exercise;” *n* = 3), and external (e.g., “I feel pressure from my family/friends to exercise;” *n* = 4) regulations, as well as amotivation (e.g., “I do not see why I have to exercise;” n = 4). Each item is rated on a 5-point Likert scale ranging from 0 = “Not true for me” to 4 = “Very true for me.” The wording of the BREQ-2 was adapted for the present study. Indeed, the introductory sentence was changed for « In general, I practice physical activity because... » and the wording of each item was adapted to refer to PA in general, instead of specific exercise sessions. These changes reduce the gap in the specificity between the psychosocial variable (motivation) and accelerometer-derived PA behavior, the latter not being specific to an intentional exercise session.

#### Accelerometer-derived PA

At T1, participants received an ActiGraph accelerometer with an elastic band, and they were ask to wear it at their waist during four consecutive weeks or 28 days, except in the water, as the device is water-resistant, but not waterproof. To retrieve the accelerometer, appointments were made with the participants four weeks after T1. Participants therefore had the accelerometer during 28 to 39 days. This triaxial accelerometer continuously measured acceleration on three axes at a rate of 30 samples per second. Data were downloaded with the ActiLife software (Version 6.13.3), which allowed the data—a movement intensity unit called “count”—to be converted into 1-min epochs for the full wear time. Using the ActiLife default cut point, 1952 counts per min or higher were considered MVPA [41]. Counts were then grouped into bouts of at least 10 min of MVPA to facilitate a comparison with PA guidelines. The non-wear period was defined according to ActiLife’s default option, a period of 60 min where no bouts are recorded, and 10 h of wear time per day was required to be considered valid [42, 43]. The median number of valid days reported was 32.

Thirty-four participants (20.48%) provided at least 21, but less than 28 valid days of accelerometric data, and 132 (79.52%) provided at least 28 valid days. This compliance rate (i.e., 79.52%) is comparable to the one found in most prior SDT-based studies using accelerometers within adult populations (i.e., between 57% and 100%) [34–38]*.* The ActiLife software provided the average number of minutes spent practicing MVPA per day from the data recorded by the ActiGraph accelerometers, which was multiplied by seven to obtain the mean Weekly MVPA used as objective PA measures for the analysis. Then, this variable was used to form two groups: one accumulating and one not accumulating at least 150 min MVPA/week. Only 30.3% of the sample engaged in enough MVPA to satisfy the guideline of 150 min per week, which corresponds to the literature on PA participation among T2D adults [6–8].

#### Acute diabetes-related symptoms

Given that only acute diabetes-related symptoms (i.e., fatigue, cognitive distress, hypoglycemia and hyperglycemia) are expected to show a change within a short period of time [28], such as over a one-month period, they were the only symptoms measured in this study. Although acute diabetes-related symptoms are not a physiological measures of glycaemia (or the glucose that circulates in the blood), they resulted from fluctuating blood sugars. Hence, the corresponding subscales of the Diabetes Symptom Checklist-Revised [44] were used to assess acute diabetes-related symptoms. This questionnaire is also considered a valid and reliable instrument for measuring the subjective severity of acute diabetes-related symptoms encountered in the past month [28, 45]. The fatigue subscale comprises four items associated with symptoms of psychological fatigue (“Lack of energy”, “An overall sense of fatigue”, “Increasing fatigue during the course of the day”, and “Fatigue in the morning when waking up”). The cognitive distress subscale comprises four items associated with symptoms of cognitive distress (“Sleepiness or drowsiness”, “Difficulty concentrating”, “Fuzzy feeling in your head (difficulty thinking clearly)”, and “Difficulty paying attention”). The hypoglycemia subscale comprises three items associated with hypoglycemia symptoms (“Moodiness”, “Irritability just before a meal”, and “Easily irritated or annoyed”). The hyperglycemia subscale comprises four items associated with hyperglycemia symptoms (“very thirsty”, “dry mouth”, “frequent need to empty your bladder”, and “drinking a lot (many beverages)”). Respondents must first confirm whether the symptom occurred in the past month (“yes” or “no”) and then, if the symptom did occur, they must indicate to what extent the symptom was troublesome on a 5-point Likert scale: 1 = “Not at all,” 2 = “A little,” 3 = “Moderately,” 4 = “Very,” and 5 = “Extremely.”

### Data analysis

All statistical analyses were performed using SPSS Statistics (version 24.0.0.0). Data were screened for missing values, multivariate outliers, and normality [46]. All variables were distributed normally. Following data cleaning, a series of analyses was performed. First, correlational analyses were used to examine the influence of age and BMI on Weekly MVPA while an analysis of variance (ANOVA) was conducted to verify the impact of sex on this same PA outcome. Second, a path analysis, adjusting for the effects of sex and BMI, was conducted to examine the relationship between each motivation, Weekly MVPA, and the level of acute diabetes-related symptom burden. Third, analyses of covariance (ANCOVAs) were performed to examine the impact of meeting PA recommendations on acute diabetes-related symptom burden. Fourth, motivation scores were transformed into z-scores [39] to perform a hierarchical cluster analysis using Ward’s method with squared Euclidian distances. Fifth, the number of clusters was determined using the agglomeration schedule coefficient and dendrogram, after which K-means clustering was implemented to confirm the cluster solution. Sixth, ANCOVAs were conducted in combination with a bootstrapping procedure to examine the impact of motivational profiles on Weekly MVPA. Finally, a chi-square analysis was performed to verify whether the proportion of adults with T2D differed significantly in terms of meeting PA recommendations when regrouped by motivational profiles.

## Results

### Descriptive analysis

Table 1 shows the descriptive statistics and correlations for the final sample (*N* = 165). Participants presented higher scores for autonomous motivation than for controlled motivation and amotivation. The results indicated that the average number of minutes spent practicing MVPA per week over the past month was 129.71 (*SD* = 115.80) and that 50 participants (30.30%) followed PA recommendations (at least 150 min of Weekly MVPA). On average, participants reported that acute diabetes-related symptoms occurred at least once in the past month and were a little troublesome to them.

**Table 1:** Descriptive statistics and correlation matrix of the sample (*N* = 165)

	α	M	SD	1	2	3	4	5	6	7	8	9.1	9.2	9.3
1. Age		61.88	8.92											
2. BMI		30.78	6.49	−0.30^***^										
3. Intrinsic regulation	0.95	4.34	1.63	0.14	−0.24^***^									
4. Identified regulation	0.80	4.98	1.16	0.21^*^	−0.25^***^	0.66^***^								
5. Introjected regulation	0.77	2.43	1.65	0.02	0.09	−0.04	0.14							
6. External regulation	0.85	1.44	1.62	−0.03	0.26^***^	−0.23^***^	− 0.22^***^	0.34^***^						
7. Amotivation	0.59	0.50	0.8	0.03	0.16^*^	−0.27^***^	−0.26^***^	0.16	0.54^***^					
8. Weekly MVPA		129.71	115.80	0.02	−0.29^***^	0.29^***^	0.11	−0.16	−0.35^***^	− 0.22^***^				
T2D acute symptoms														
9.1. Fatigue	0.82	2.05	1.03	−0.15	0.17^*^	−0.23^***^	− 0.16^*^	0.04	0.01	−0.10	− 0.26^***^			
9.2. Cognitive distress	0.81	1.80	0.92	−0.15	0.09	−0.22^*^	− 0.23^***^	0.00	− 0.02	−0.10	− 0.20^*^	0.75^***^		
9.3. Hypoglycemia	0.78	1.67	0.88	−0.06	0.01	−0.12	− 0.12	0.07	0.04	0.01	−0.07	0.44^***^	0.56^***^	
9.4. Hyperglycemia	0.81	1.97	1.00	0.07	0.16^*^	−0.09	−0.08	0.09	0.10	0.09	−0.19^*^	0.40^***^	0.53^***^	0.50^***^

Weekly MVPA; over the entire period the accelerometer was worn, average number of minutes spent practicing MVPA per week

^*^p < 0.05, ^***^p < 0.001

### Motivation, PA behavior and acute diabetes-related symptoms

Results of correlational analyses showed no significant association between age and Weekly MVPA (*r =* 0.02, *p* = 0.78). Thus, age was not considered a control variable in subsequent analyses. However, results of an ANOVA revealed that sex was significantly associated with Weekly MVPA [F (1, 163) = 21.07, *p* < 0.001], while results of correlational analyses showed that BMI was significantly related to this same PA outcome (*r =* − 0.29, *p* < 0.001) as well as with fatigue (*r =* 0.17, *p* < 0.05) and hyperglycemia (*r =* 0.16, *p* < 0.05) symptoms. Therefore, sex and BMI were included as control variables in the path analysis.

Results of the path analysis (see Table 2) demonstrated that intrinsic and external regulations were significantly associated with Weekly MVPA, which, in turn, was significantly related to the “fatigue”, “cognitive distress” and “hyperglycemia” symptoms. The model presented satisfactory fit indices: χ2 (24, *n* = 165) = 33.07, *p* = 0.10, RMSEA = 0.05 (90% C.I. = 0.00–0.09), CFI = 0.97, TLI = 0.95, SRMR = 0.05 [47, 48]. More specifically, findings showed that: 1) intrinsic regulation was positively associated with Weekly MVPA (*B* = 0.16, *p* < 0.001) while external regulation was negatively associated with this same PA outcome (*B* = − 0.16, *p* < 0.001), 2) identified and introjected regulations, as well as amotivation, were not significantly associated with Weekly MVPA, 3) Weekly MVPA was negatively associated with fatigue (*B* = − 0.22, *p* < 0.001), cognitive distress (*B* = − 0.19, *p* < 0.001), and hyperglycemia (*B* = − 0.16, *p* = 0.01) symptoms. Moreover, the indirect effects of intrinsic and external regulations on fatigue, cognitive distress, and hyperglycemia symptoms through Weekly MVPA were all significant (*p* < 0.05). Specifically, intrinsic regulation was indirectly and negatively associated with lower fatigue, cognitive distress and hyperglycemia symptoms, through Weekly MVPA (all *p <* 0.05) while the opposite was true for external regulation (all *p* < 0.05).

**Table 2:** Path analysis of the relationship between motivation, weekly MVPA, and acute T2D symptoms (N = 165)

Outcome	Predictor	B	S.-E.	*Β*	Bootstrapped CI 95%
Motivation = > Weekly MVPA					
Weekly MVPA	Sex	−65.89	16.18	−0.57^***^	[− 0.82, − 0.32]
	BMI	−2.91	1.27	−0.03^*^	[− 0.05, 0.00]
	Intrinsic regulation	18.12	5.42	0.16^***^	[0.07, 0.24]
	Identified regulation	−16.17	9.27	−0.14	[−0.24, − 0.07]
	Introjected regulation	−1.90	5.64	−0.02	[−0.17, 0.10]
	External regulation	−17.88	5.34	−0.16^***^	[−0.29, 0.01]
	Amotivation	−3.96	7.81	−0.03	[−0.11, 0.08]
Weekly MVPA = > acute T2D symptoms					
Fatigue	BMI	0.02	0.05	0.02	[−0.01, 0.04]
	Weekly MVPA	−0.00	0.00	−0.22^***^	[−0.33, − 0.12]
Cognitive distress	BMI	0.01	0.05	0.01	[−0.02, 0.03]
	Weekly MVPA	−0.00	0.00	−0.19^***^	[−0.29, − 0.09]
Hypoglycemia	BMI	−0.00	0.02	−0.00	[− 0.02, 0.02]
	Weekly MVPA	0.00	0.00	−0.07	[−0.20, 0.06]
Hyperglycemia	BMI	0.02	0.05	0.02	[−0.01, 0.04]
	Weekly MVPA	−0.00	0.00	−0.16^*^	[−0.28, − 0.03]
Indirect effects of motivation on acute T2D symptoms via Weekly MVPA ^a^					
Fatigue	Intrinsic regulation	−0.03	0.01	−0.04^*^	[− 0.06, − 0.01]
	External regulation	0.03	0.01	0.04^*^	[0.01, 0.06]
Cognitive distress	Intrinsic regulation	−0.03	0.01	−0.03^*^	[− 0.05, − 0.01]
	External regulation	0.03	0.01	0.03^*^	[0.01, 0.05]
Hyperglycemia	Intrinsic regulation	−0.02	0.01	−0.03^*^	[−0.05, 0.00]
	External regulation	0.02	0.01	0.03^*^	[0.00, 0.05]

*Note.* Results of an ANOVA showed that sex was significantly associated with Weekly MVPA while results of a correlational analysis showed that BMI was related to this same outcome as well as to the overall T2D related symptoms. Therefore, the path analysis was controlling for sex and BMI. Bootstrapping procedures with a sample size of 10,000 was used following Chernick’s (2007) recommendations to examine indirect effects. Weekly MVPA: over the entire period the accelerometer was worn, average number of minutes spent practicing MVPA per week. CI; confidence interval

^a^Only significant indirect effects are reported

* *p* < 0.05, *** *p* < 0.001

In addition to the path analysis, ANCOVAs (see Table 3) were performed to verify whether participants meeting PA recommendations in terms of Weekly MVPA (achieving at least 150 min of Weekly MVPA over the past month) would experience less acute diabetes-related symptom burden over the past month. As results of correlational analyses showed that BMI was significantly related to fatigue and hyperglycemia symptoms, the ANCOVAs were controlling for participants’ BMI. Since the largest group was less than four times larger than the smallest one (a ratio 2.3), no formal homogeneity of variance test was required [46]. With a bootstrapping procedure using a resample of 3000 [49], results of four ANCOVAs showed that participants meeting PA recommendations also reported significantly (Bonferroni adjusted) less burden associated with fatigue [F (1, 162) = 5.83, *p* < 0.05], cognitive distress [F (1, 162) = 7.43, *p* < 0.01], and hyperglycemia [F (1, 162) = 4.77, *p* < 0.05] symptoms over the past month. The difference was not significant for hypoglycemia symptoms.

**Table 3:** Impact of meeting PA recommendations on acute diabetes-related symptoms (*N* = 165)

				Meeting PA recommendations					
				No (*n* = 115)		Yes (*n* = 50)			
Acute diabetes-related symptoms	Univariate F-value (1, 162)	Bonferonni adjusted *p*	Eta^2^	M	SD	M	SD	Difference (SE)	Bootstrapped CI 95%
Fatigue (*R*^*2*^_*adj*_ = 0.05)	5.83	0.01	0.04	2.02	0.99	1.58	0.72	0.39 (0.14)	[0.11, 0.65]
Cognitive distress (*R*^*2*^_*adj*_ = 0.04)	7.43	0.00	0.04	1.90	1.01	1.45	0.59	0.43 (0.13)	[0.18, 0.69]
Hypoglycemia (*R*^*2*^_*adj*_ = 0.00)	2.04	0.13	0.01	1.54	0.79	1.36	0.61	0.18 (0.12)	[−0.06, 0.42]
Hyperglycemia (*R*^*2*^_*adj*_ = 0.04)	4.77	0.02	0.03	2.00	0.98	1.60	0.72	0.35 (0.15)	[0.06, 0.64]

Note. The four ANCOVAs were controlling for BMI given that results from the correlation matrix showed that it was significantly associated with fatigue and hyperglycemia symptoms. Meeting PA recommendations: achieving at least an average of 150 min of MVPA per week. Bootstrapping procedures with a sample size of 3000 was used following Chernick’s (2007) recommendations

### Motivational profiles and PA behavior

#### Cluster analysis: Two-step procedure

Results of the hierarchical cluster analysis showed one drastic increase in the agglomeration schedule, demonstrating that only one solution matched the data. More specifically, compared to the three previous changes in the agglomeration schedule (8.56%, 13.09%, and 14.48%), a greater increase was found when four clusters merged to three (21.75%). Thus, the four-cluster solution was deemed suitable, as large increases imply the merging of dissimilar clusters [50]. Following the hierarchical cluster analysis, k-means clustering was used to confirm the four-cluster solution. The composition of the final cluster solution was examined according to sex, age, and BMI using a chi-squared test of independence and two ANOVAs, respectively. The results showed that motivational profiles did not differ in terms of sex [χ2 (3, *n* = 165) = 0.75, *p* = .86] or age [F (3, 161) = 0.69, *p* = 0.56], but that they did in terms of BMI [F (3, 161) = 4.51, *p* < 0.01]. Using a Bonferroni correction, results of post-hoc tests showed that participants from the Self-Determined profile (*M* = 28.87) displayed a significantly lower BMI score than those from the Low Motivation (*M* = 32.48) and Non-Self-Determined (*M* = 33.09) profiles. Therefore, BMI was included as control variable in subsequent analyses with motivational profiles.

**Fig. 1:**
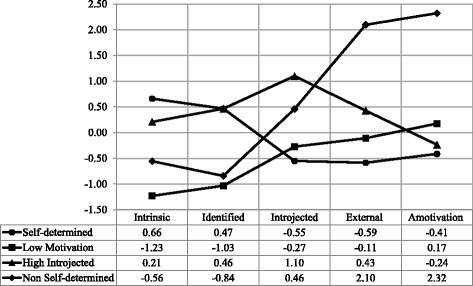
Motivational profiles toward PA

Figure 1 shows the four motivational profiles. Based on prior SDT-based research on motivational profiles, clusters were labeled: Self-Determined, High Introjected, Low Motivation, and Non-Self-Determined. The Self-Determined and Non-Self-Determined clusters were named according to prior SDT studies on T2D adults’ motivational profiles [18, 19]. The Self-Determined cluster comprised 43.64% (*n* = 72) of participants, who displayed the highest scores for intrinsic and identified regulations and lower scores for introjected and external regulations and amotivation. The Non-Self-Determined cluster comprised 8.48% (*n* = 14) of participants, who presented the highest scores for amotivation, lower scores for intrinsic and identified regulations, and moderate scores for introjected and external regulation. Because neither Gourlan et al. [19] nor Castonguay and Miquelon [18] found similar clusters in their studies, the other two clusters were named based on prior SDT studies examining motivational profiles among adults from the general population. Specifically, the Low Motivation cluster, comprising 23.64% (*n* = 39) of the participants who obtained low scores for each motivation and moderate scores for amotivation, was named based on motivational profiles from previous SDT-based studies conducted among adults from the general population [51–54] which found a similar cluster (i.e., participants presenting low scores for each motivation, except amotivation, for which they scored higher). Finally, the fourth cluster was labeled High Introjected because its characteristics were similar to a profile reported by Stephan, Boiché and Le Scanff [55] in a study of adults from the general population. These authors found that participants from this cluster presented high scores for introjected regulation, moderate scores for intrinsic and identified regulations, and low scores for external regulation. High introjected participants (24.24% or *n* = 40) presented high scores for introjected regulation; moderate scores for intrinsic, identified, and external regulations; and low scores for amotivation.

#### Effects of motivational profiles on PA behavior

First, a one-way ANCOVA, controlling for the effects of sex and BMI, was conducted to examine how each cluster solution or motivational profile was associated with Weekly MVPA over the past month. Following recommendations from Tabachnick and Fidell [46], a formal homogeneity of variance test was required, because the largest group was 5.14 times larger than the smallest group, exceeding the 4 to 1 criterion. A Levene’s test was thus used for Weekly MVPA and its result was not significant (*p* > 0.05). Homogeneity of variance was therefore assumed. To verify further the normality of the residuals, a Shapiro–Wilk test was used, which found significance (*p* < 0.001). Because bootstrapping is a non-parametric test making no assumptions in terms of distribution, including normality [56], the ANCOVAs were performed with a bootstrapping procedure using a resample of 3000 [49]. The results are presented in Table 4, indicating that cluster membership significantly explained 24% of the variances in Weekly MVPA [F (3, 159) = 4.35, *p* < 0.01]. Using a Bonferroni correction, the results of post-hoc tests showed that, compared to participants from other clusters, participants from the Self-Determined profile displayed the highest score on Weekly MVPA (*M* = 167.91), followed by participants from the High Introjected (*M* = 114.54), Low Motivation (*M* = 101.54), and Non-Self-Determined (*M* = 55.01) clusters. Apart from the difference between the Low Motivation and the High Introjected clusters, all differences between clusters were significant (all *p* < 0.05).

**Table 4:** Impact of motivational profiles on Weekly MVPA over a one-month period (N = 165)

PA behavior	Univariate F-value (3, 159)	Eta^2^	Motivational Profiles Mean (SD)	Posthoc (Bonneferonni)	Difference (SE)	Bootstrapped 95% CI
Weekly MVPA (*R*^*2*^_*adj*_ = 0.22)	4.35^**^	0.24	Self-Determined	vs Low Motivation	50.29^**^ (18.22)	[14.93, 86.08]
			167.91 (129.33)	vs High Introjected	47.11^*^ (21.27)	[5.08, 88.67]
				vs Non-Self-Determined	93.29^***^ (23.15)	[46.59, 139.00]
			High Introjected	vs Low Motivation	3.19 (18.98)	[−32.53, 41.19]
			114.54 (116.61)	vs Non-Self-Determined	46.18^*^ (22.83)	[3.81, 92.96]
			Low Motivation	vs Non-Self-Determined	43.00^*^ (20.71)	[2.46, 84.16]
			101.54 (76.39)			
			Non-Self-Determined			
			55.01 (59.09)			

*Note.* Results of an ANOVA showed that sex was significantly associated with Weekly MVPA while results of a correlational analysis showed that BMI was also related to this same outcome. Therefore, the ANCOVA was controlling for sex and BMI. Bootstrapping procedures with a sample size of 3000 was used following Chernick’s (2007) recommendations. Weekly MVPA; over the entire period the accelerometer was worn, average number of minutes spent practicing MVPA per week

* *p* < 0.05, ** *p* < 0.01, *** *p* < 0.001

The relationship between motivational profiles and meeting PA guidelines in terms of Weekly MVPA (achieving at least 150 min of Weekly MVPA over the past month) was examined using a chi-square test (see Table 5). Results revealed that the chi-square value was significant, [*χ*^2^ (*dl* = 3, *N* = 165) = 9.88, *p* < 0.05], indicating variances in meeting PA guidelines among the motivational profiles. However, results showed a moderate association [Cramer’s *V* = .25, *p* < .05] [57]. As shown in Table 5, the Self-Determined profile showed a significantly higher proportion of participants currently meeting MVPA guidelines and a significantly lower proportion of participants not currently meeting MVPA guidelines. Thus, the group with the highest proportion of participants accumulating at least 150 min of Weekly MVPA also presented a Self-Determined profile. The opposite was true for the Non-Self-Determined profile, which was associated with the highest proportion of participants not accumulating at least 150 min of Weekly MVPA.

**Table 5:** Relationship between motivational profiles and meeting PA recommendations for T2D management over a one-month period (N = 165)

Motivational profiles		Observance of PA recommendations for adults with T2D		χ^2^	Cramer’s *V*
		Yes	No		
Self-Determined	Observed frequency	30 (41.7%)^b^	42 (58.3%)^a^	9.88^*^	.25^*^
	Expected frequency	21.8	50.2		
High Introjected	Observed frequency	11 (27.5%)^b^	29 (72.5%)^b^		
	Expected frequency	12.1	27.9		
Low Motivation	Observed frequency	8 (20.5%) ^b^	31 (79.5%)^b^		
	Expected frequency	11.8	27.2		
Non-Self-Determined	Observed frequency	1 (7.1%)^b^	13 (92.9%)^a^		
	Expected frequency	4.2	9.8		

*Note.* Observing 150 min of MVPA: achieving at least an average of 150 min of MVPA per week. When comparing the “Yes” and “No” column, different letters indicate significant differences between observed and expected frequency in term of observance of PA recommendations (e.g. for the group who is not observing 150 min MVPA/week, the self-determined profile has an observed frequency that is significantly lower than expected and for the group who is observing 150 min MVPA/week, the self-determined profile has an observed frequency that is significantly higher than expected). Bootstrapping procedures with a sample size of 3000 was used following Chernick’s (2007) recommendations

*p < 0.05

## Discussion

The objective of this study was to examine, over a one-month period, how PA motivation would influence accelerometer-derived PA behavior, and ultimately, acute diabetes-related symptom burden among T2D adults. Using both a person- and variable-centered approach, this objective was attain by means of: 1) investigating the indirect effect of PA participation on the relationship between PA motivation and acute diabetes-related symptom burden among participants and 2) examining whether participants who met PA recommendations (i.e., 150 min of moderate-to-vigorous PA per week) would experience less acute diabetes-related symptom burden over a one-month period. Consistent with statistics indicating less than 35% of the adult population with diabetes (type 1 and 2) meets PA recommendations [6–8], the results herein reveal 30.30% of participants were accumulating at least 150 min of Weekly MVPA, while 69.69% were not.

While a large body of literature used the SDT framework to study PA among adults from the general population (see [11] for a review), few studies have focused on T2D adults [12–19], and even fewer have adopted a person-centered approach for a T2D population [18, 19], which considers how each motivation type combines to influence PA behavior. This study uses a person-centered approach and compares it to a variable-centered approach to examine the impact of PA motivation on PA behavior among T2D adults. So far, it is the first SDT-based study assessing PA behavior among T2D adults using accelerometers. Therefore, its main originality resides in the investigation of the link between PA motivation and motivational profiles, as conceptualized by SDT, and accelerometer-derived PA, including meeting PA recommendations, in a sample of T2D adults. Another important contribution is the examination of the indirect effect between PA motivation and acute diabetes-related symptoms through weekly PA participation.

### Motivation, PA behavior and acute diabetes-related symptoms

In regards of the relationship between PA motivation and accelerometer-derived PA behavior, results of the path analysis demonstrated that, over a one-month period, intrinsic motivation was positively associated with the average number of minutes spent practicing MVPA per week, whereas external regulation was negatively associated with this same PA outcome. However, introjected regulation was not significantly associated with Weekly MVPA, corroborating the results of the few prior studies that surveyed the relationship between this type of motivation and subjective PA behavior among T2D adults [17–19] or accelerometer-derived PA behavior among non-T2D adult populations [29, 31, 32]. Findings also revealed that although the link between amotivation and PA behavior was negative, it was insignificant. While, based on prior SDT-based research [18, 19], this latter result was unexpected, similar results were reported in past SDT-based studies conducted among non-T2D populations, into which PA behavior was assess subjectively [11] or objectively [29, 30, 32].

Overall, these findings underscore the importance of considering the role of both intrinsic and extrinsic motivations in terms of PA participation among T2D adults, which is consistent with the results of past SDT-based studies using self-report PA measures (e.g., [12, 13, 18, 58, 59]), but less consistent with the findings of studies using objective PA measures [30–33]. More specifically, while the majority of the aforementioned studies conducted with an objective PA measure reveals a positive relationship between intrinsic regulation and PA behavior, none show a significant link between external regulation and PA behavior. Therefore, and given that past literature using self-report PA measure has often found both significant and insignificant results in regards of the relationship between external regulation and PA behavior [11], more research is needed to determine if the nature of PA measure (objective instead of subjective) is a key element influencing the significance of this relationship.

As to the relationship between accelerometer-derived PA behavior and diabetes-related symptom burden, results showed that the average number of minutes spent practicing MVPA per week was negatively associated with “fatigue”, “cognitive distress” and “hyperglycemia” symptoms. However, since, apart from the herein study, no research has looked at the relationship between PA and diabetes-related symptom burden in T2D adults, more studies are needed to investigate this issue over a longer period (e.g., 2 months). Finally, results of the path analysis also revealed significant indirect effects between intrinsic as well as external regulation and acute diabetes-related symptoms through Weekly MVPA, thus supporting a mediation effect [60]. Accordingly, the herein findings propose that, over a one-month period, the more T2D adults engage in PA for intrinsic motives, the more time they spent practicing MVPA per week, which, in turn, is associated with less acute diabetes-related symptoms burden. Conversely, they imply that the more T2D adults engage in PA for extrinsic motives, the less time they spend practicing MVPA per week, which, in turn, is associated with more acute diabetes-related symptoms burden. Thus, given its significant influence on PA participation, the quality of PA motivation should be consider when it comes to achieving better glycemic control and experiencing less diabetes-related symptoms burden among T2D adults.

In addition to the path analysis’s findings, results of the ANCOVAs showed that, over a one-month period, participants meeting PA recommendations or practicing at least 150 min of Weekly MVPA, also reported experiencing less acute diabetes-related symptom burden. These results suggest that, over a month period, meeting PA recommendations should be associated with less fluctuating blood sugars, and therefore less acute diabetes-related symptom burden. Nevertheless, more studies are needed to explore this relationship using both a measure of acute diabetes-related symptom burden and an objective measure of blood glucose fluctuation, obtained by means of a glucometer.

### Motivational profiles and PA behavior

Four motivational profiles emerged from the results: Self-Determined, High Introjected, Low Motivation, and Non-Self-Determined. Participants from the Self-Determined profile mainly engage in PA because the activity is enjoyable, while participants from the High Introjected profile engage in moderate PA for all motivations, while tending to exhibit higher introjected (e.g., guilt) motives. Participants from the Low Motivation profile engage in moderate PA for all motivations, with a tendency toward amotivation. Finally, participants from the Non-Self-determined profile tend to be prominently amotivated or externally motivated toward PA participation, suggesting they engage in PA for mainly external motives or without motivation. Notably, the past literature on PA motivational profiles among T2D adults found profiles similar to the Self-Determined and Non-Self-Determined profiles [18, 19]. However, because no study conducted with T2D adults found profiles similar to the High Introjected or Low Motivation profile, these were named based on the results of previous work with non-T2D adult populations [51–55]. Worth mentioning is that although 43.64% of the sample presented a Self-Determined profile, this proportion is consistent with past research (e.g. [19, 51–54, 61]) conducted among populations both with and without T2D.

Findings revealed that the Self-Determined profile was associated with the highest score for number of minutes spent practicing MVPA per week over the past month. Inversely, and as expected, the lowest scores for this PA outcome were associated with the Non-Self-determined profile. However, the Low Motivation and High Introjected profiles reported similar moderate PA participation scores in terms of Weekly MVPA and meeting PA guidelines (i.e., their PA outcome scores did not differ significantly). The absence of a difference between these two later profiles suggests that higher scores on both autonomous and controlled motivation types of are not necessarily associated with higher scores on PA participation. In fact, regarding PA participation, the most beneficial motivational profile is the one with a higher level of autonomous motivation and a lower level of controlled motivation. Therefore, to promote PA participation, autonomous motivation should be as high as possible, while controlled motivation should be as low as possible. In agreement with prior work on T2D adults’ motivational profiles [18, 19], these findings suggest that increasing both autonomous and controlled motivation types would not necessarily lead to more PA participation. Some differences were also found to be marginally significant in terms of PA participation among other motivational profiles. The Weekly MVPA score obtained by participants from the Non-Self-Determined profile was marginally lower than that obtained by participants from the High Introjected profile. Although these results are marginally significant, they concur with the observation that profiles with higher autonomous motivation and lower controlled motivation are associated with greater PA participation.

In sum, the results obtained regarding motivational profiles are consistent with those of prior studies that used self-report measures to assess PA behavior [18, 19], as the highest rate of PA participation was found among participants from the Self-Determined profile, while the lowest rate was found among participants from the Non-Self-determined profile. These findings support the assumption that a higher level of autonomous motivation is beneficial to PA engagement among T2D adults, while a higher level of controlled motivation can be detrimental. They also suggest that the beneficial impact of autonomous motivation on PA is dependent on the level of controlled motivation and underscore the importance of considering all motivation types and their combination when assessing T2D adults’ PA participation.

### Implications of findings

The results of this study stress the importance of using both a variable- and person-centered approach when examining the impact of motivation on PA participation among T2D adults. A variable-centered approach first allows for the examination of which motivation type has the greatest impact on PA participation. Then, using a person-centered approach has the advantage of allowing for the consideration of the multiple PA motivations reported by individuals. For instance, the results show that while Self-Determined and High Introjected profiles present similar levels of autonomous motivation, the High Introjected profile is associated with lower scores for PA participation, which could not have been determined using a variable-centered approach. The herein results also demonstrate that PA motivation, as conceptualized by SDT, is associated with accelerometer-derived PA participation, which is associated with lower acute diabetes-related symptoms burden. These findings certainly highlight the importance of considering PA motivation when it comes to T2D management. They show that while any motivation can encourage someone to be more active, autonomous motivations should be promoted, while controlled ones should be lessened in order to promote PA participation.

The results also have practical implications, with the most essential being the importance of educating T2D adults and their health practitioners about the importance of PA-related motivation types. As shown in the results, autonomous motives are positively related to PA participation and meeting PA guideline, while controlled motives are negatively linked to these same outcomes, individually or in the form of motivational profiles. At first, T2D adults may find it difficult to engage in PA for fun or because they appreciate its related benefits. They may be more inclined to engage in PA to comply with their doctor’s recommendations. Although this latter reason represents a controlled form of motivation, it still contributes to the initiation of the behavior and is thus not entirely detrimental to PA participation. Furthermore, it is possible to transition from a controlled to an autonomous motivation. In time, individuals can learn and experience the benefits of PA or discover facets of the activity they did not know they would enjoy. In doing so, individuals can change their attitudes toward PA and engage in PA for more autonomous motives [62]. That said, the results obtained regarding motivational profiles underline that it may not be sufficient to promote solely autonomous types of motivation. Specifically, the findings related to motivational profiles emphasize the importance for T2D adults’ and health professionals’ awareness of the unfavorable impact of controlled motivation and amotivation on PA participation. In agreement with this later observation, considering motivational profiles and their influence on PA behavior (using both a variable- and a person-centered approach) could lead health professionals to achieve greater improvement in PA participation among T2D adults*.*

### Limitations, strengths, and future research

Some limitations warrant a cautious interpretation of this study’s conclusions. First, despite using a prospective longitudinal design, it is nevertheless inappropriate to make causal inferences, because causality cannot be established with correlational data. To do so would require an experimental design. Second, given the number of participants (*n* = 165), this study would have benefited from a larger sample size. Indeed, SDT-based studies that have used PA objective measures with populations other than T2D adults [30, 32, 33] generally reported a small (*r* = 0.10) to medium (*r* = 0.30) effect size [63] for the relationship between motivation types and PA behavior. Through these studies, identified and introjected regulations were found to be significantly associated with objective PA behavior. Because the ability of an analysis to detect an effect is dependent on both the size of said effect and the sample size, it is possible to believe that the weak and non-significant relationship reported in the present study could have been significant given a larger sample size. For instance, in the present study, using G*Power 3.1.9.2 [64, 65] and assuming a power of 0.80 and α of 0.05, with a correlation coefficient of 0.11, a sample size of 646 would have been necessary to detect a significant effect between identified regulation and Weekly MVPA.

Third, as participants were recruited year-round, those who participated during the winter may have been less prone to engage in PA than those who participated during the summer. However, if connected online periodically, the GT3X accelerometer allows for continuous data recording during a virtually infinite period. Thus, future studies can control for this limitation by monitoring participants’ PA behavior for 12 consecutive months. A forth limitation, also associated with the use of accelerometers, is that some participants reported practicing aquatic activities. As the GT3X is water-resistant, but not waterproof, participants had to remove the device in such situations. Therefore, using a waterproof accelerometer or a waterproof case would strengthen the PA measure. In addition, given that the accelerometer was worn at the participants’ waists only, data may not be representative of PA not primarily involving waist movement (e.g., upper-body resistance training, cycling, and rowing) [66–68]. Moreover, the herein study reports the average number of minutes spent practicing MVPA per week and whether this PA was performed in the context of intentional sessions (e.g., leisure time PA). Thus, while a PA self-report measure would have allowed for a distinction with regard to the PA context (e.g., leisure, work or transport related), the accelerometers do not, which is an important limitation of the present study. Hence, future studies planning to assess PA with an objective measure should consider ways to distinguish PA contexts.

A fifth limitation was that acute diabetes-related symptom distress was self-reported. While this measure provides useful insights regarding fluctuating blood sugars over a one-month period, it would be essential for future research to include an objective measure of blood sugar fluctuation. For instance, in addition to the DSC-R, researchers could require participants to report their daily glucose levels using a continuous glucose monitoring system, allowing for live remote glucose monitoring. More specifically, a glucose sensor worn by the patient could send glucose level readings to the patient’s cellphone via Bluetooth, which would in turn be sent to a centralized server at the researcher’s facility via the Internet. This way, researchers could monitor participants’ blood glucose levels from a remote location (e.g., lab or office), as long as everyone has access to the Internet [69]. At last, another limitation of this study lies in the potential lack of generalizability associated with results of the cluster analysis, given that these findings are sample specific, which could explain the difficulty in replicating the clusters found in other studies conducted with T2D adults. However, the profiles reported in the herein study present characteristics similar to those usually reported among adults with T2D or the general population (e.g. [18, 19, 55]). The difficulty in replicating the ‘High Combined profile’ found by Gourlan, Trouilloud and Boiché [19] or the ‘Moderate and Controlled profile’ found by Castonguay and Miquelon [18] highlights the importance for future research to study motivational profiles in adults with T2D in diverse and larger samples*.*

Despite these limitations, the present study provides useful insights into PA motivation and PA behavior among T2D adults. For instance, it offers a unique contribution to SDT-based research on motivation and PA behavior among T2D adults that examines the impact of both motivation types and profiles on accelerometer-derived PA. It also contributes to research on PA behavior and diabetes management by providing results regarding the link between accelerometer-derived PA behavior and acute diabetes-related symptoms. Because individuals tend to overvalue their PA participation and undervalue their sedentary time when using self-report measures, and because this discrepancy increases even more with higher levels of PA [70], one of the major strengths of this work is its use of an objective assessment of PA behavior (accelerometers).

## Conclusion

Compared to T2D adults who do not meet PA recommendations and therefore engage in less Weekly MVPA, T2D adults who meet PA recommendations are more inclined to engage in PA for enjoyment. These results highlight the importance of promoting autonomous motives for PA adoption and maintenance among T2D adults. Moreover, the findings also suggest that meeting PA recommendations for T2D could influence the incidence of acute diabetes-related symptoms, further supporting the importance of T2D adults’ PA motives. Given the actual need to understand better why so few T2D adults regularly practice PA, despite its beneficial effects on health, the present findings support the importance of studying the quality of the motivation associated with PA behavior in T2D adults.

## Abbreviations

ANCOVA: Analysis of covariance
ANOVA: Analysis of variance
BMI: Body Mass Index
BREQ-2: Behavioral Regulation in Exercise Questionnaire-version 2
MVPA: Moderate-to-vigorous physical activity
PA: Physical activity
SD: Standard deviation
SDT: Self-Determination Theory
T2D: Type 2 diabetes

## Footnotes

1 While the mean age of participants (62.05 years old) is high, it is still representative of the T2D adult population; therefore, the generalizability of the results is not threatened. In fact, 90% of adults diagnosed with diabetes in the US are 45 years old or older, with almost half being 65 or older [71]. The proportions are similar for Canadians [72, 73].
